# Developing an *E. coli* heterologous expression system for characterizing a marine debrominase from *Roseobacter* sp.

**DOI:** 10.1128/aem.01980-25

**Published:** 2026-04-03

**Authors:** Xiaofang Li, Sen Yang, Yuping Liu, Hua Huang, Xinshuai Zhang, Yin Zhong, Lorenz Adrian, Ping’an Peng

**Affiliations:** 1 Guangdong Provincial Key Laboratory of Biotechnology for Plant Development, School of Life Sciences, South China Normal University 12451, Guangzhou, China; 2 State Key Laboratory of Advanced Environmental Technology, Guangzhou Institute of Geochemistry, Chinese Academy of Sciences 74556 , Guangzhou, China; 3 University of Chinese Academy of Sciences 74519, Beijing, China; 4 Shenzhen Readline Biotech CO., Ltd., Shenzhen, China; 5 Guangdong Key Laboratory of Environmental Protection and Resources and Utilization, Guangzhou, China; 6 Guangdong-Hong Kong-Maco Joint Laboratory for Environmental Pollution and Control, Guangzhou, China; 7 Department of Molecular Environmental Biotechnology, Helmholtz Centre for Environmental Research — UFZ 28342, Leipzig, Germany; 8 Chair of Geobiotechnology, Technische Universität Berlin 26524, Berlin, Germany; Shanghai Jiao Tong University, Shanghai, China

**Keywords:** reductive dehalogenase, debrominase, *Roseobacter*, *Escherichia coli*, heterologous expression

## Abstract

Reductive dehalogenases (RDases) are a family of enzymes that employ a cobamide cofactor and two iron-sulfur clusters to selectively cleave carbon-halogen bonds. The identification of yet-uncharacterized RDases is crucial for advancing our understanding of dehalogenation processes in both natural and engineered environments, particularly for bioremediation. In this study, we developed a heterologous expression system in *Escherichia coli* DH5α to produce a chloroform-reducing RDase (TmrA) from *Dehalobacter* sp. UNSWDHB, using a pTrcHisA vector without requiring an additional vector for vitamin B_12_ transporters. By co-expressing the pRSFDuet-BtuCEDFB plasmid, we further enhanced active TmrA production without relying on the T7 promoter system. Using this system, we achieved the first heterologous expression of an RDase (the debrominase Ros_A3X954) from a marine heterotrophic *Roseobacter* strain, without chemical cofactor reconstitution. Ros_A3X954 catalyzes conversion of 2,6-dibromophenol to 2-bromophenol with a specific activity of 21.8 ± 1.0 nmol s^−1^ mg^−1^. The enzyme exhibited optimal activity at pH 6.0 and demonstrated significant thermal stability by maintaining full catalytic capacity across a broad temperature range (20–50°C), a key advantage for applications in thermally fluctuating marine environments. These findings expand our understanding of RDase functional diversity in common marine microbiota and highlight the significant application potential of *Roseobacter* for bioremediation of bromophenol pollution.

**IMPORTANCE:**

Organohalide-respiring bacteria (OHRB) play a crucial role in bioremediation by degrading halogenated pollutants using cobamide-dependent reductive dehalogenases (RDases). While these enzymes hold great promise for bioremediation, their practical application has been limited by difficulties in production and heterologous expression. This study successfully developed an *Escherichia coli* DH5α expression system for RDases without requiring vitamin B_12_ transporters expression vector, significantly simplifying production process. A bromophenol-degrading RDase (Ros_A3X954) from *Roseobacter* sp. was discovered, exhibiting high activity at pH 6.0 and thermal stability (20–50°C). This study not only provides a scalable platform for RDase exploration but also highlights *Roseobacter*’s ecological role in bromine cycling, advancing bioremediation strategies for persistent pollutants.

## INTRODUCTION

Organohalides play pivotal roles in a wide range of applications (1–3). For example, chlorinated ethenes, chloroform (CF), and bromophenols have been widely used as solvents, cleaners, and defoaming agents (1, 2). Beyond anthropogenic sources, organohalides also occur naturally, with their discovery having increased exponentially over the past five decades (4, 5). While organohalides offer significant benefits in applications, the inherent stability of carbon–halogen bonds renders them persistent in the environment, leading to widespread contamination. Organohalide-respiring bacteria (OHRB) have emerged as a promising solution. Under anoxic conditions, OHRB can utilize toxic organohalides as respiratory electron acceptors. Consequently, microbial reductive dehalogenation is regarded as a practical approach for organohalides remediation (6–9).

Reductive dehalogenases (RDases) are a family of redox enzymes essential for anaerobic organohalide respiration (10). These oxygen-sensitive, redox-active enzymes are typically characterized by the presence of a cobamide cofactor in the active center and two iron-sulfur clusters, which are essential for their catalytic activity (11). RDases can be broadly divided into two categories: respiratory RDases and catabolic RDases (12). Respiratory RDases are expressed with a twin arginine translocation (TAT) signal peptide that relocate them to the periplasmic space, where they function as terminal reductases in the respiratory chain. In contrast, catabolic RDases lack a TAT signal peptide, remain in the cytosol, and generally exhibit greater oxygen tolerance (13). Despite their importance, only a limited number of RDases have been successfully purified from their native producers, often requiring labor-intensive procedures to obtain sufficient biomass and to isolate the enzymes (14–16). To address these challenges, researchers have explored heterologous expression systems as an alternative approach. For instance, the tetrachloroethene (PCE) RDase (PceA) from *Desulfitobacterium hafniense* was heterologously expressed in *Shimwellia blattae*, a non-dechlorinating but cobamide-producing bacterium (17). Similarly, a chloroform RDase (TmrA) from *Dehalobacter* sp. was expressed in *Bacillus megaterium*, a naturally cobamide-producing strain, under the control of a xylose-inducible promoter (18). Moreover, *Bacillus megaterium* was employed in the heterologous expression of NpRdhA, a non-respiratory RDase from *Nitratireductor pacificus* strain pht-3B (13). However, these systems often yielded low quality of the target enzyme, limiting their practical utility.

*Escherichia coli* is a promising host for the heterologous expression of RDases owing to its rapid growth, high protein productivity, compatibility with modern molecular biology tools, and suitability for large-scale cultivation and downstream processing (16, 19, 20). For example, PceA from *Dehalobacter restrictus* was expressed in *E. coli* through the fusion with a trigger factor protein which was cleaved off after expression; the resulting enzyme lacked catalytic activity but could be activated by chemical cofactor reconstitution under anoxic conditions (19). In another study, a vinyl chloride RDase (VcrA) was expressed as a fusion protein with maltose-binding protein in *E. coli,* but the enzyme was inactive and precipitated in inclusion bodies; again, subsequent chemical cofactor reconstitution enabled the enzyme to catalyze the reduction of vinyl chloride, dichloroethenes, and 1,2-dichloroethane (20). In summary, most of the early approaches produced inactive enzymes and required chemical reconstitution.

Recent advances in the heterologous expression of RDases in *E. coli* have focused on optimizing cobalamin uptake and enhancing enzyme solubility to avoid the chemical reconstitution requirement (12, 21–23). Picott et al. demonstrated the use of the pBAD42-BtuCEDFB plasmid in various *E. coli* strains with enhanced [4Fe-4S] cluster formation activity to improve the heterologous expression of TmrA, an RDase from *Dehalobacter* sp. UNSWDHB that catalyzes the transformation of CF to dichloromethane (DCM) (12). Their findings highlighted that the co-expression of the vitamin B_12_ salvage pathway (*btu*) operon, combined with TmrA induction under anoxic conditions, is critical for the production of catalytically active RDases. Further extending this approach, Ng and Silver employed the same expression system to screen and identify 36 RDases originating from diverse microbial strains capable of dechlorinating PCE and/or deiodinating the thyroid-disrupting compound 2,4,6-triiodophenol (21). In an innovative study, a self-sufficient RDase system—which requires no external redox partner proteins for dehalogenation—was developed in *E. coli*. This system facilitates the extraction and transfer of low-potential electrons derived from pyruvate, thereby driving reductive dehalogenation of brominated and iodinated phenolic compounds using NADPH as an electron donor (22).

Overall, the majority of heterologously expressed RDases tend to be inactive, primarily due to the shortage of cobamide cofactor and/or the misfolding of the protein backbone. The choice of plasmids and host strains with different copy number/promoters may affect the activity of heterologously expressed RDases. For instance, although the commonly used T7 promoter can strongly increase the overall protein yield, it does not necessarily enhance protein solubility or specific activity. Therefore, careful screening of plasmids and host strains is essential to establish a more efficient system for producing active RDases. *E. coli* possesses a natural cobalamin salvage pathway via the *btu* system, which transports cobalamin across the cell membrane and thereby supports cobalamin incorporation into heterologously expressed proteins (24, 25). In a previous study, we demonstrated that *E. coli* strain DH5α combined with the pTrcHisA plasmid is suitable for expressing active metalloenzymes (26). However, it remains to be determined whether the *E. coli* DH5α/pTrcHisA system supports the expression of functionally active RDases.

In this study, we extended previous studies by screening various plasmid and promoter combinations in *E. coli* for the expression of active RDase. We found that *E. coli* DH5α carrying the pTrcHisA-TmrA vector, even without an additional vector for vitamin B_12_ transporters, could yield active TmrA. We further optimized the expression system by employing both pTrcHisA-RDase and pRSFDuet-BtuCEDFB plasmids in *E. coli* DH5α. This system showed higher efficiency than previously reported T7 promoter-dependent systems in *E. coli* BL21(DE3), as first demonstrated using TmrA expressed without a T7 promoter. Using this optimized platform, we screened for active RDases and identified a previously uncharacterized enzyme (Ros_A3X954) from *Roseobacter* sp., which exhibited dehalogenation activities against 2,6-dibromophenol (2,6-DBP). The *Roseobacter* group represents a predominant lineage in global oceans, where environments are rich in both natural and anthropogenic organohalides such as 2,6-DBP (27–30). The presence of RDase genes in these predominantly aerobic, yet metabolically versatile bacteria points to a previously unrecognized capacity for organobromide transformation. This study not only establishes a robust heterologous expression platform for reductive debrominases but also highlights the potential ecological role of *Roseobacter* sp. in marine bromine cycling.

## RESULTS

### Expression of TmrA in *E. coli* with different plasmids

**Fig 1:**
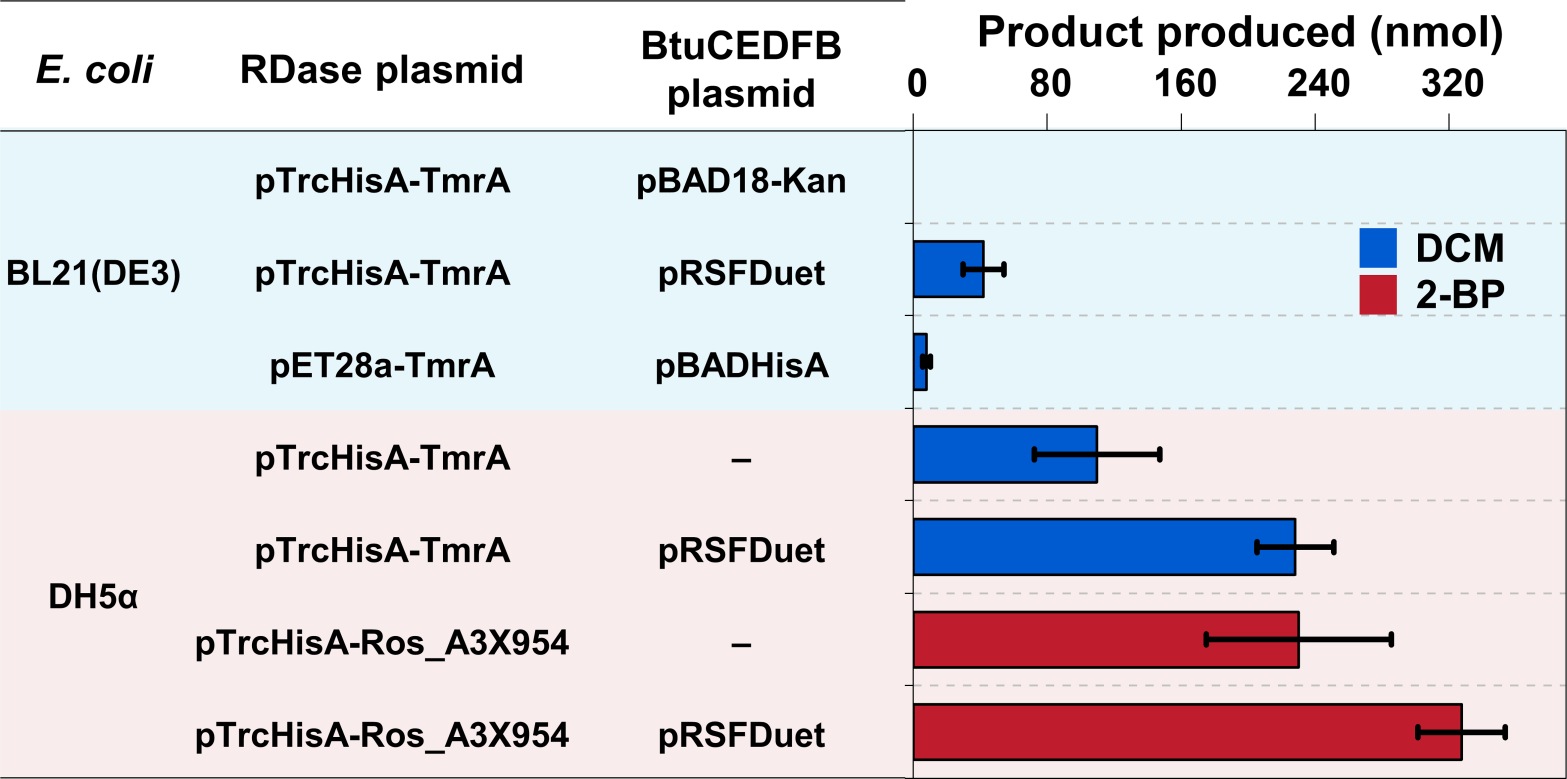
Reduction of CF to DCM by TmrA and of 2,6-DBP to 2-BP by Ros_A3X954. The RDases were heterologously expressed in two *E. coli* isolates carrying different RDase-plasmids and Btu-plasmids. Reduction of CF and 2,6-DBP was achieved through an overnight incubation with cell lysates. Data from three independent replicates are presented.

The expression of functionally active TmrA from a *Dehalobacter* strain, as previously reported by Picott et al. (12), was successfully reproduced (Fig. 1). In this procedure, TmrA was co-expressed with the proteins of a *btu* operon-encoded vitamin B_12_ transporters in *E. coli* BL21(DE3) under anoxic conditions, using a T7 promoter system. The formation of DCM from CF served as a marker for confirming the expression of active TmrA (12). We constructed three distinct plasmid combinations for co-expression: (i) pET28a-TmrA with pBADHisA-BtuCEDFB, (ii) pTrcHisA-TmrA with pRSFDuet-BtuCEDFB, and (iii) pTrcHisA-TmrA with pBAD18-Kan-BtuCEDFB. These plasmids are standard vectors for heterologous expression in *E. coli* and were readily available from our laboratory resources. The main differences in the chosen plasmids are the promoters from which expression occurs: pET28a-TmrA and pRSFDuet-BtuCEDFB use the strong T7 promoter but rely on the presence of the T7 polymerase in the host strain, pTrcHisA-TmrA uses the strong Trc promoter that is independent of the T7 polymerase, and pBADHisA-BtuCEDFB and pBAD18-Kan-BtuCEDFB use the arabinose promoter described to express with lower strength than T7 and Trc promoters. Notably, the combination of pET28a-TmrA and pRSFDuet-BtuCEDFB was excluded due to incompatibility issues arising from shared antibiotic resistance markers.

Activity tests with the heterologously expressed RDases revealed distinct outcomes among plasmid combinations (Fig. 1). Although active TmrA was successfully produced in *E. coli* BL21(DE3) with either the pET28a-TmrA/pBADHisA-BtuCEDFB or pTrcHisA-TmrA/pRSFDuet-BtuCEDFB plasmid combination, the pTrcHisA-TmrA/pRSFDuet-BtuCEDFB system exhibited higher enzymatic activity, resulting in a sixfold higher formation of DCM from the dechlorination of CF. This difference may result from the higher copy number of pRSFDuet (reported by the supplier to have a copy number of >100 copies per cell) that increased expression levels of the Btu transporter system. The pTrcHisA-TmrA/pBAD18-Kan-BtuCEDFB co-expression system failed to generate detectable TmrA activity.

### Screening for uncharacterized RDases

We employed a Sequence Similarity Network (SSN) approach to select candidate RDases from UniProt databases for characterization (Fig. S1). Eight putative RDases were randomly selected from clusters with no reported dehalogenation activity, including Q3ZA21 (from *Dehalococcoides mccartyi* 195), Q3Z6A6 (from *Dehalococcoides mccartyi* 195), A0A2J1DV62 (from *Dehalococcoides mccartyi* H1-3-2.001), A0A0C6EJ57 (from *Dehalococcoides* sp. UCH007), A0A2J1DXP0 (from *Dehalococcoides mccartyi* H1-3-2.001), Ros_A3X954 (from *Roseobacter* sp. MED193), A0A2E6VL94 (from *Actinomycetes bacterium*), and A0A0S2I3V8 (from *Salinivirga cyanobacteriivorans* L21-Spi-D4). We utilized the expression system consisting of *E. coli* BL21(DE3) with pTrcHisA and pRSFDuet-BtuCEDFB plasmids to assess the activity of TmrA and eight uncharacterized RDases against a range of organohalides, including CF, PCE, 2,6-DBP, trichloroethene (TCE), 1,1-dichloroethane (1,1-DCA), and 1,1,1-trichloroethane (1,1,1-TCA) (Table S1). The results reveal that the cell lysates containing TmrA had no catalytic activity toward any of the tested organohalides except CF. However, a previous study demonstrated that purified TmrA could dechlorinate 1,1,1-TCA, with a maximum specific activity approximately four times lower than that observed for CF (31). No dechlorination product of 1,1,1-TCA was detected in the presence of TmrA in our study, which may be attributed to several factors: (i) the relatively low expression efficiency in *E. coli* BL21(DE3) carrying the pTrcHisA and pRSFDuet-BtuCEDFB plasmids may have compromised enzymatic activity; (ii) the use of cell-free lysates in the activity assay could lead to an underestimation of the enzyme’s specific activity; and (iii) 1,1,1-TCA exhibits greater resistance to dechlorination compared to CF. Among the uncharacterized RDases, only the cell lysates containing Ros_A3X954 demonstrated dehalogenation activity (Table S2). Ros_A3X954 can transform 2,6-DBP to 2-bromophenol (2-BP) (Fig. S2).

### Optimization of the host strains of the expression system

To investigate host-dependent expression characteristics, we analyzed TmrA production (measured by DCM formation from CF) in *E. coli* DH5α with pTrcHisA-TmrA and pRSFDuet-BtuCEDFB plasmids. Comparative quantification revealed a fivefold increase in DCM formation in *E. coli* DH5α vs BL21(DE3) carrying the same plasmid combination (Fig. 1), confirming that *E. coli* DH5α was the more effective expression host for this system.

To delineate the functional role of pRSFDuet-BtuCEDFB, parallel experiments with *E. coli* DH5α lacking this plasmid revealed baseline TmrA activity at 50% of the full-system yield (Fig. 1). This twofold increase demonstrates the pRSFDuet-BtuCEDFB plasmid’s capacity to amplify active TmrA production. A similar enhancing effect was observed for Ros_A3X954, whose debromination activity (measured by 2-BP formation from 2,6-DBP) was significantly higher in the presence of the pRSFDuet-BtuCEDFB plasmid.

Notably, *E. coli* DH5α with the pTrcHisA-TmrA plasmid showed 14-fold higher TmrA activity than *E. coli* BL21(DE3) carrying the pET28a-TmrA/pBADHisA-BtuCEDFB system. This is likely because the T7 expression system in BL21(DE3) often generates substantial inclusion bodies for such enzymes, yielding high total protein but low soluble and active enzyme. Conversely, the moderate expression strength of the pTrcHisA plasmid makes it more suitable for obtaining active RDases.

### Dehalogenation activity of heterologously expressed TmrA and Ros_A3X954

**Fig 2:**
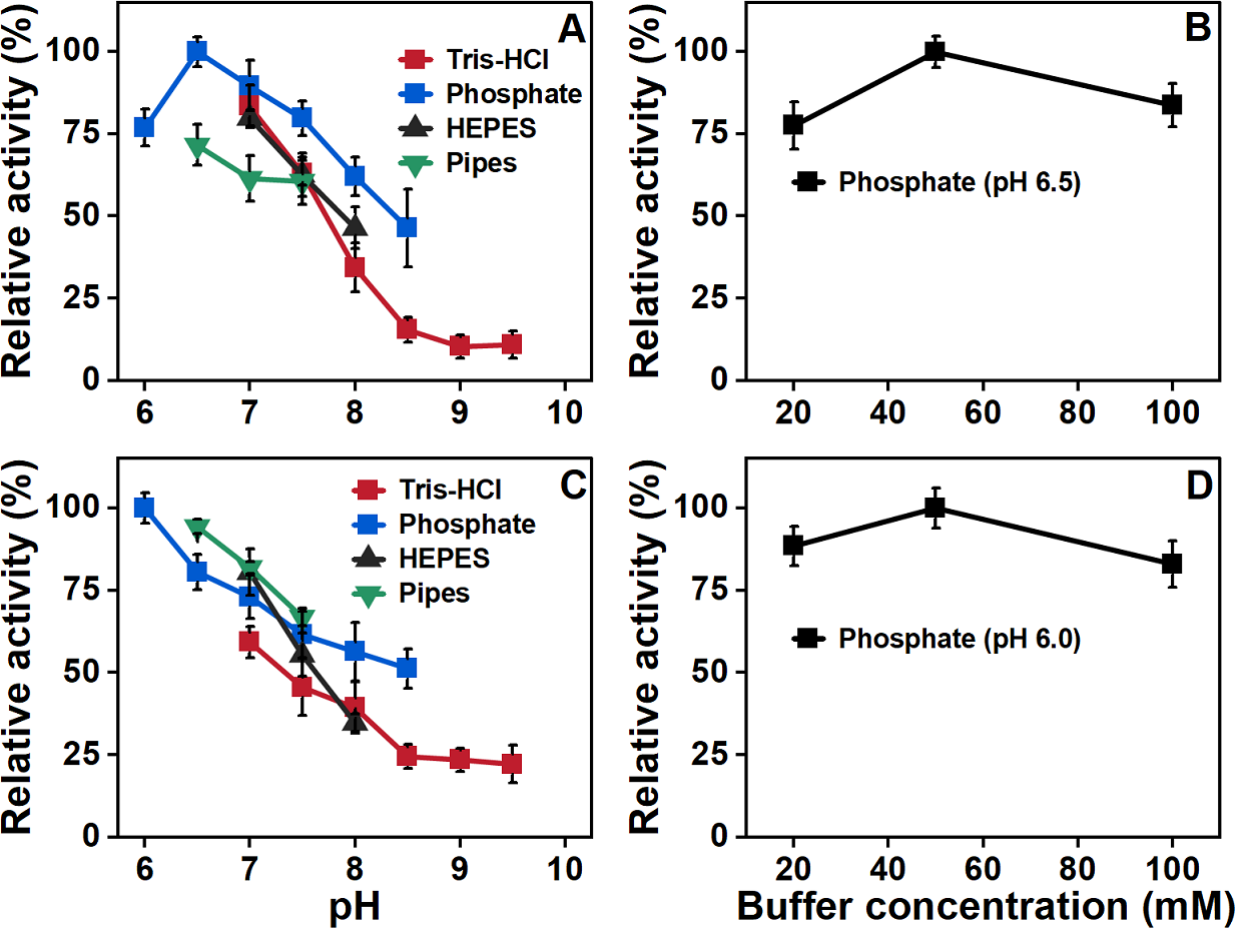
The effect of pH and buffer concentration on the activity of TmrA (**A and B**) and Ros_A3X954 (**C and D**). Assays were conducted in four different buffer systems across a range of pH (**A and C**) and in an optimized buffer with varying concentrations (**B and D**). Purified TmrA and Ros_A3X954 were heterologously expressed in *E. coli* DH5α with the pRSFDuet-BtuCEDFB plasmid and assessed for their respective catalytic activities: conversion of CF to DCM and of 2,6-DBP to 2-BP at 25°C. The enzyme activity was quantified from the products (DCM and 2-BP) formed after incubation with purified enzymes. The relative activity for each panel is presented as a percentage, normalized to the maximum activity observed under its specific tested conditions (defined as 100%).

To quantify the dehalogenation activities of TmrA and Ros_A3X954, kinetic data were collected using purified enzymes co-expressed in DH5α with the pRSFDuet-BtuCEDFB plasmids. TmrA and Ros_A3X954 were purified using a Ni-NTA resin column, yielding a final concentration of 5.37 mg mL^−1^ and 1.35 mg mL^−1^, respectively. The influence of buffer conditions on the kinetic parameters was systematically investigated (Fig. 2). TmrA and Ros_A3X954 demonstrated the highest catalytic activity in sodium phosphate buffer. For TmrA, the catalytic activity in the conversion of CF to DCM in sodium phosphate buffer increased as the pH rose from 6.0 to 6.5 but declined as the pH further increased from 6.5 to 8.5. The optimal buffer concentration and pH for TmrA were determined to be 50 mM and 6.5, respectively. In the case of Ros_A3X954, its catalytic activity exhibited a decrease in dehalogenation activity as the buffer pH increased, with optimal activity observed at pH 6.0 in 50 mM sodium phosphate buffer.

**Fig 3:**
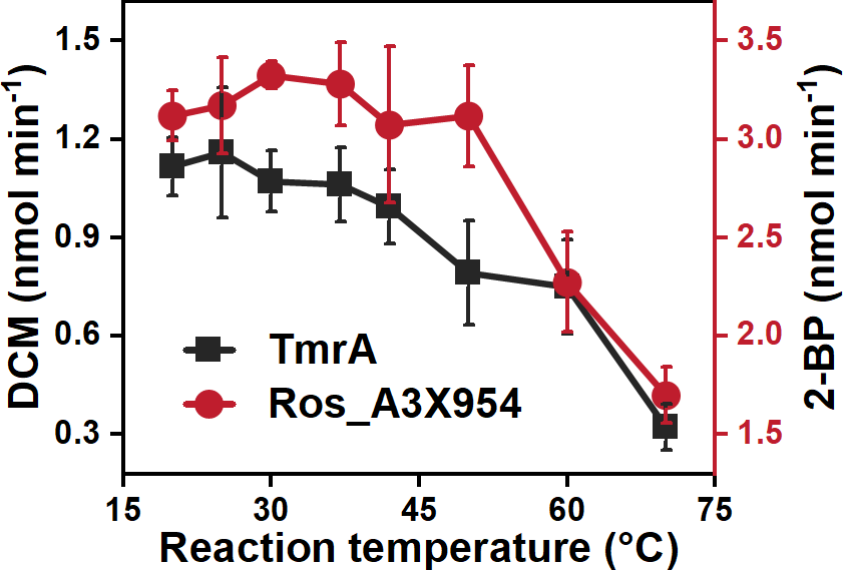
Thermal stability of TmrA and Ros_A3X954. Purified TmrA and Ros_A3X954 were heterologously expressed in *E. coli* DH5α with the pRSFDuet-BtuCEDFB plasmid and assessed for their respective catalytic activities: conversion of CF to DCM and of 2,6-DBP to 2-BP at 25°C. Data from three independent replicates are presented.

The influence of temperature on the dehalogenation activities of TmrA and Ros_A3X954 was systematically evaluated (Fig. 3). TmrA achieved its maximum activity at 25°C, with a gradual decline in activity observed as temperatures rose above this point. In contrast, Ros_A3X954 demonstrated excellent thermal stability, maintaining consistent activity after the enzyme was incubated at 50°C for 5 min. The oxygen-tolerance experiments revealed that TmrA experienced a decline in activity following exposure to oxygen and exhibited a half-life of 60 min under oxygen exposure (Fig. S3). In contrast, Ros_A3X954 was highly sensitive to oxygen, losing its dehalogenation activity upon oxygen exposure (data not shown).

**Fig 4:**
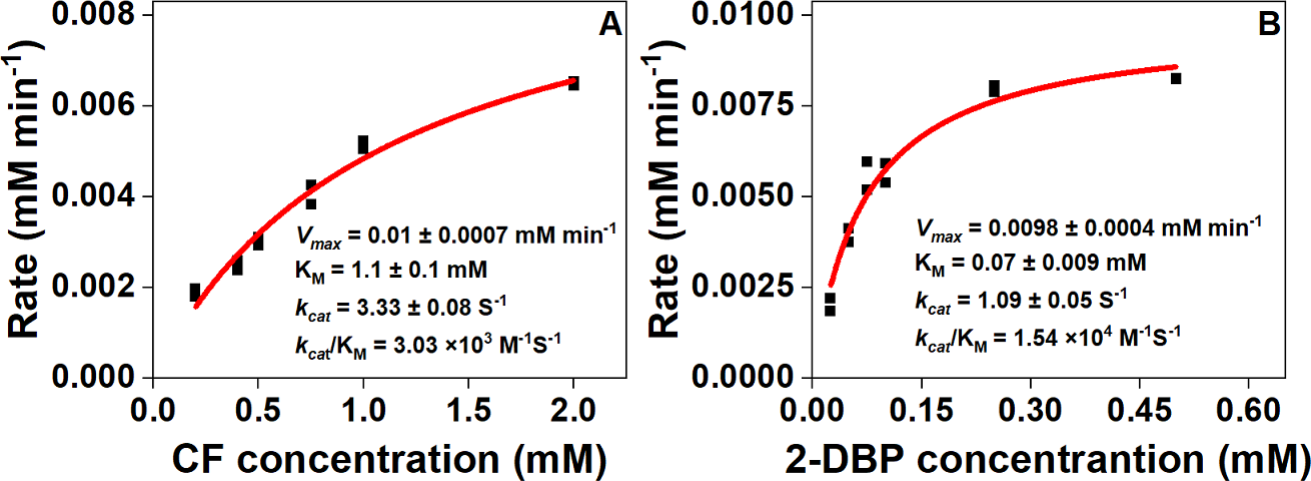
Kinetic curves for purified TmrA (**A**) and Ros_A3X954 (**B**). The initial concentration of TmrA and Ros_A3X954 were 0.05 μΜ and 0.15 μΜ, respectively. Initial velocities at different substrate concentrations were measured and then fitted to the Michaelis-Menten equation using OriginPro 2024 software.

TmrA dechlorinated CF with a *k*_cat_ of 3.3 ± 0.08 s^−1^ and a *k*_cat_/*K*_M_ of 3.0 × 10^3^ M^−1^ s^−1^ (Table 1; Fig. 4). The specific activity of TmrA was 55.6 ± 3.9 nmol s^−1^ mg^−1^, approximately 20 times lower than the activity observed for TmrA co-expressed in *E. coli ara-sufΔisc* with the p15TVL and the *btu* plasmids (31). Ros_A3X954 exhibited a *k*_cat_ of 1.1 ± 0.05 s^−1^, a *k*_cat_/*K*_M_ of 1.5 × 10^4^ M^−1^ s^−1^, and a specific activity of 21.8 ± 1.0 nmol s^−1^ mg^−1^ for the debromination of 2,6-DBP to 2-BP (Table 1; Fig. 4).

**TABLE 1:** Kinetic parameters for RDases expressed in *E. coli*

Enzyme	*E. coli*	Dehalogenation	*k*_cat_ (s^−1^)	*K*_M_ (μM)	*V*_max_ (μM min^−1^)	Specific activity (nmol s^−1^ mg^−1^)	*k*_cat_/*K*_M_ (M^−1^ s^−1^)	Reference
TmrA^*a*^	DH5α	CF to DCM	3.3 ± 0.08	1,000 ± 100	10 ± 0.7	55.6 ± 3.9	3.0 × 10^3^	This study
Ros_A3X954^*a*^	DH5α	2,6-DBP to 2-BP	1.1 ± 0.05	71 ± 9	10 ± 0.4	21.8 ± 1.0	1.5 × 10^4^	This study
JtRdhA	HMS174(DE3)	2,6-DBP to 2-BP			10 ± 1			(22)
TmrA	*ara-sufΔiscR*	CF to DCM	53 ± 8	61 ± 14		1,100 ± 160	8.7 × 10^5^	(31)
TmrA	BL21(DE3)pLysS^*b*^	CF to DCM				0.98 ± 0.57		(32)
RdhC1	BL21(DE3)*ΔiscR*	2,4,6-TBP to phenol^*c*^				0.12 ± 0.02		(21)

^*a*^ Purified TmrA and Ros_A3X954 were heterologously expressed in *E. coli* DH5α with the pRSFDuet‑BtuCEDFB plasmid.

^*b*^ Heterologous expression of TmrA in* E. coli* followed by cofactor reconstitution.

^*c*^ 2,4,6-TBP represents 2,4,6-tribromophenol.

### Cofactor characterization of the two expressed RDases

The molar iron (Fe) content was determined to be 9.52 ± 0.03 mol per mol TmrA and 8.06 ± 0.8 mol per mol Ros_A3X954, which were expressed in *E. coli* DH5α with pTrcHisA and pRSFDuet-BtuCEDFB plasmids (Table S3). Similarly, the acid-labile sulfur (S) content was measured as 10.7 ± 0.06 mol per mol TmrA and 5.64 ± 0.8 mol per mol Ros_A3X954. The calculated RDase:Fe:S ratios of 1:9.5:10.7 (TmrA) and 1:8:5.6 (Ros_A3X954) closely approximate the theoretical ratio of 1:8:8, suggesting two [4Fe-4S] clusters per enzyme. Additionally, cobalt levels were quantified as 0.83 ± 0.02 mol per mol TmrA and 0.11 ± 0.003 mol per mol Ros_A3X954, respectively, indicating cobalt incorporation into their catalytic centers. The low cobalt content in Ros_A3X954 may be attributed to the inefficient incorporation of the cobamide cofactor during folding in *E. coli*, a process that occurs co-translationally and is variable among different RDases and expression systems; one mol of the active RDase proteins contains one mol of Co. Therefore, the percentage of the active RDases among the total produced TmrA and Ros_A3X954 proteins expressed using *E. coli* DH5α with pTrcHisA and *btu* plasmids system was calculated to be 83% and 11%, respectively.

For enzymes expressed without pRSFDuet-BtuCEDFB plasmids, the calculated RDase:Fe:S ratios of 1:4.4:3.5 (TmrA) and 1:3.5:3.9 (Ros_A3X954), and the cobalt (Co) content was quantified as 0.35 ± 0.003 mol per mol TmrA and 0.06 ± 0.0007 mol per mol Ros_A3X954, respectively. Co-expressed with BtuCEDFB in the pTrcHisA/DH5α system increased the incorporation of Co, Fe, and S into both RDases. Previous studies have shown that cobalamin plays a critical role in iron-sulfur cluster maturation because of the close contact between cobalamin and the proximal [4Fe-4S] cluster (13, 23). The higher cobalamin incorporation achieved with BtuCEDFB is likely responsible for the elevated Fe and S contents and the more complete assembly of [4Fe-4S] clusters in these enzymes.

### Phylogenetic analysis of the functionally expressed RDases

**Fig 5:**
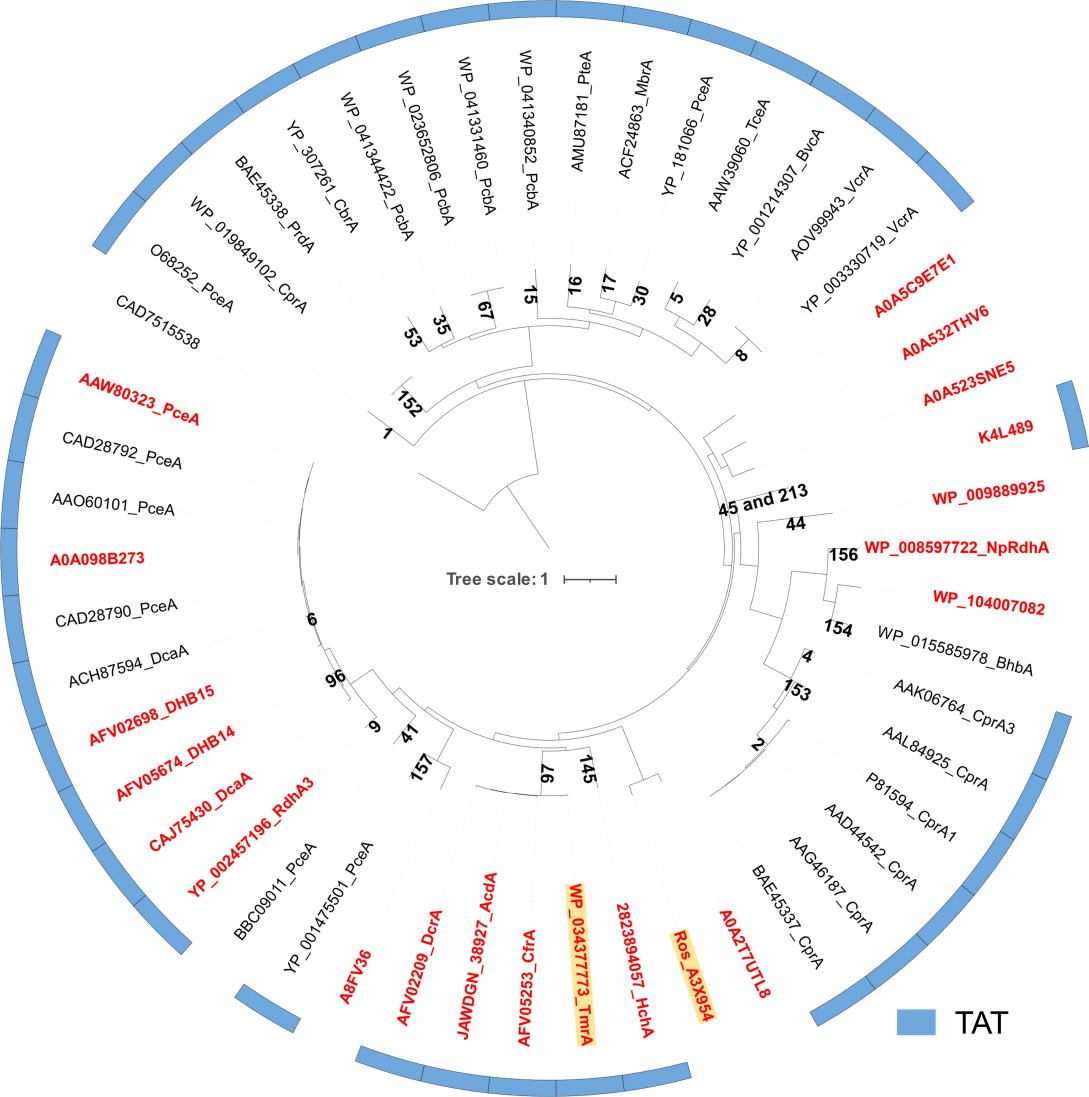
Phylogenetic tree of functionally characterized RDases. Labels in red indicate RDases that have been heterologously expressed and shown to retain catalytic activity, whereas others were characterized in their native hosts. Orange highlighting denotes RDases heterologously expressed in this study. An outer blue ring identifies RDases containing a TAT signal peptide. Epoxyqueuosine reductase (CAD7515538) was used as the outgroup. Orthologous group numbers of RDases are labeled on the corresponding clades.

A phylogenetic tree was constructed using 51 functionally characterized RDases, with orthologous groups (OGs) assigned (Fig. 5). Among these, 21 RDases have been characterized through heterologous expression. The heterologously expressed and functionally active RDases include those from *Dehalobacter* (K4L489, 2823894057, WP_034377773, AFV05253, JAWDGN_38927, AFV02209, AFV05674, and AFV02698), *Desulfitobacterium* (YP_002457196, CAJ75430, A0A098B273, and AAW80323), *Roseobacter* sp. (Ros_A3X954), *Pararhodobacter* (A0A2T7UTL8), *Jhaorihella thermophila* (WP_104007082), *Nitratireductor pacificus* (WP_00859772), *Clostridioides* (WP_009889925), *Shewanella* (A8FV36), *Candidatus Bathyarchaeota archaeon* (A0A523SNE5), *Candidatus Lokiarchaeota archaeon* (A0A532THV6), and *Thorarchaeota archaeon* (A0A5C9E7E1) (Fig. 5) (12, 13, 17, 18, 21, 22, 31, 33). Notably, among these bacteria, only *Dehalobacter* is an obligate OHRB. A key distinction is that RDases from *Dehalobacter* and *Desulfitobacterium* contain TAT signal peptides, whereas RDases from other taxa lack them. Ros_A3X954, a catabolic RDase that lacks a TAT signal peptide, harbors a reductive dehalogenase domain (PF13486) spanning residues 174–269 and contains two [4Fe-4S] cluster-binding motifs located at positions 283–294 (CDICTKCADACP) and 337–345 (CAICMRVCP). Ros_A3X954 shares 72.2% identity with A0A2T7UTL8 (from *Pararhodobacter aggregans*), which has been reported to reduce 2,4,6-triiodophenol to phenol (21). Interestingly, Ros_A3X954 was not assigned to any OGs in the Reductive Dehalogenase Database (34). These findings highlight the unique characteristics of Ros_A3X954 within the RDase enzyme family.

## DISCUSSION

Bioremediation with dehalogenase-containing organisms is an effective strategy for mitigating organohalide contamination in the environment. However, the structural and functional characterization of most RDases remains elusive due to challenges in their separation and preparation, which has significantly impeded the application of both RDases and OHRB in bioremediation efforts (22, 31). To accelerate the functional verification of RDases, we established an optimized heterologous co-expression system in *E. coli* DH5α using the pTrcHisA vector, which operates independently of the T7 promoter. This system enhanced TmrA activity 14-fold compared to the conventional T7-based pET28a/BL21(DE3) system, underscoring a fundamental trade-off in heterologous expression: the T7 system increases raw protein yield but often at the expense of functional activity for complex metalloenzymes, as overexpressed enzymes frequently misfold or fail to incorporate cofactors correctly. The pTrcHisA/DH5α system may circumvent this issue by providing moderate, T7-free expression that favors the production of soluble, active enzyme, a strategy that has been successfully applied to other challenging enzymes such as xanthine oxidoreductases (26).

Notably, when supplemented with the pRSFDuet-BtuCEDFB plasmid, the pTrcHisA/DH5α system achieved a further increase in efficiency, yielding a 29-fold enhancement over the T7-based system. This suggests that the endogenous promoter of the *b*tuCEDFB operon and the high copy number of the pRSFDuet plasmid may contribute significantly to this effect. However, this hypothesis requires direct experimental validation, and the underlying mechanism warrants further investigation.

Previous studies by Picott et al. explored various *E. coli* strains—such as BL21(DE3)*ΔiscR*, BL21(DE3) Lobstr, *SufFeScient,* and *ara-sufΔisc*—in conjunction with a *btu* plasmid to express active TmrA. These strains (with the exception of *E. coli* BL21(DE3)Lobstr) have been engineered to enhance the expression of the *isc* and/or *suf* operons, thereby promoting the production of [4Fe-4S] clusters and facilitating the heterologous co-expression of TmrA. In our study, the specific activity of TmrA co-expressed in *E. coli* DH5α with the pET28a and the *btu* plasmids is approximately 20 times lower than the activity observed for TmrA co-expressed in *E. coli ara-sufΔisc* with the p15TVL and the *btu* plasmids (31). This discrepancy in enzyme activity may be attributed to differences in host strains (*E. coli* DH5α vs *E. coli ara-sufΔisc*), leading to variations in [4Fe-4S] cluster biosynthesis, which serves as an essential cofactor for RDase activity. It should be noted, however, that *E. coli ara-sufΔisc* is a *Suf*-deficient mutant not commercially available. Although TmrA activity is lower when expressed in *E. coli* DH5α, this strain is widely used in laboratory settings and offers greater accessibility. In this study, pET28a was employed as the expression vector for TmrA in lieu of p15TVL. It remains unclear whether p15TVL could yield higher levels of TmrA enzymatic activity, thus necessitating further comparative analyses in *E. coli* DH5α.

*E. coli* DH5α harboring the pTrcHisA vector and the pRSFDuet-BtuCEDFB plasmid exhibited TmrA expression with specific activity levels 2 to 55 times higher than those of purified TmrA derived from native *Dehalobacter*, as well as heterologously expressed TmrA in corrinoid-producing *Bacillus megaterium* and in *E. coli* following cofactor reconstitution (18, 32, 35). These findings suggest that *E. coli* DH5α with the pTrcHisA vector and pRSFDuet-BtuCEDFB plasmid represents a promising system for the heterologous expression of RDases, providing significant potential for the identification and characterization of previously uncharacterized RDases.

In this study, we successfully expressed one novel debrominase, Ros_A3X954 from *Roseobacter* sp., with or without the pRSFDuet-BtuCEDFB plasmid in *E. coli* DH5α. The observed *V*_max_ values for Ros_A3X954 in the debromination of 2,6-DBP were approximately 10 μM min^−1^, comparable to the performance of JtRdhA from *Jhaorihella thermophila*, which was heterologously expressed in *E. coli* HMS174(DE3) with the *btu* plasmid (22). Ros_A3X954 demonstrates thermal stability, maintaining robust activity within a temperature range of 20°C to 50°C. This stability under elevated temperatures highlights its potential for bioremediation in elevated-temperature environments, such as industrial sites and underground water.

Phylogenetic tree analysis of functionally characterized RDases revealed that Ros_A3X954 is a catabolic RDase lacking the TAT signal peptide and not yet classified into an identified OG. Notably, Ros_A3X954 occupies a distinct cluster within the phylogenetic tree, sharing 72.2% amino acid sequence identity with A0A2T7UTL8 from *Pararhodobacter aggregans*, a known RDase that catalyzes the reduction of 2,4,6-triiodophenol to phenol (21). The integration of SSN analysis with heterologous expression is an effective strategy for elucidating the functions of uncharacterized RDase clusters. This approach not only aids in identifying novel RDases but also expands our understanding of their versatile roles in the reduction of diverse organohalides, offering valuable insights for bioremediation and biotechnological applications.

Ros_A3X954 originates from *Roseobacter* sp., a marine bacterium belonging to the *Roseobacter* lineage, a group widely distributed in marine ecosystems and capable of constituting up to 25% of marine microbial communities (36). Chen et al. (37) identified a gene, *bhbA*, which encodes a RDase that catalyzes the debromination of 3,5-dibromo-4-hydroxybenzoate to 4-hydroxybenzoate via the intermediate 3-bromo-4-hydroxybenzoate. Putative *bhbA* homologs are widely distributed among members of the *Roseobacter* clade (including the genera of *Roseobacter*). This discovery highlights the potential role of *Roseobacter* sp. in the debromination of brominated compounds in marine environments. Bromophenols are abundant in marine ecosystems due to anthropogenic activities or their synthesis by various marine organisms (5, 28–30). Sim et al. have reported that the concentration of bromophenols in marine sediments was 10–500 times higher than that in the riverine sediments (30). Bromophenols may be actively metabolized by *Roseobacter* sp., suggesting its involvement in marine bromine cycling.

Overall, this study has successfully established a robust methodology for the heterologous expression of RDase. It provides valuable insights into the substrate specificity and catalytic efficiency of debrominases from *Roseobacter* sp., thereby illuminating their potential applications in the bioremediation of environments contaminated with brominated compounds. These findings underscore the importance of exploring marine-derived enzymes for addressing pollution challenges and highlight the significant potential of RDases as versatile tools for environmental cleanup.

## MATERIALS AND METHODS

### Materials

Gene sequences (Table S4) were downloaded from NCBI and the DNA stretches with 6*×*His tag were synthesized by General Biosystems (Anhui, China). *E. coli* BL21(DE3) and *E. coli* DH5α were purchased from China General Biosystems (Anhui, China). The plasmids pTrcHisA, pET28a, pBADHisA, pBAD18-Kan, and pRSFDuet were purchased from Novagen (Darmstadt, Germany). pTrcHisA is a high copy number plasmid that is designed for strong and inducible expression of N-terminally 6*×*His-tagged proteins in *E. coli*. pBADHisA, pBAD18-Kan, and pRSFDuet plasmids were used as vectors to construct the *btu* plasmid. NEB Q5 DNA polymerase and NEB T4 DNA ligase were used for target gene amplification and DNA ligation, respectively. The Basic Seamless Cloning and Assembly Kit for homologous recombination was purchased from Beijing Quanshijin Biotechnology Co., Ltd. (Beijing, China). Plasmids and PCR products were extracted with the plasmid mini-extract kit from TIANGEN BIOTECH Co., Ltd. (Beijing, China). DNA sequencing was conducted by Sangon Biotech Co., Ltd. (Shanghai, China). Protein concentrations were determined with a Nanodrop 2000 (Thermo) at 280 nm. The optical densities (OD) of *E. coli* cultures were determined at 600 nm using a UV-2600 UV-Vis Spectrophotometer (Shimadzu, Japan). Hydroxocobalamin was purchased from Yingxin Laboratory Equipment Co., Ltd. (Shanghai, China). Other chemical reagents were purchased from Aladdin (Shanghai, China) or Millipore Sigma (Darmstadt, Germany). All anoxic experiments were conducted in a vinyl anaerobic chamber (Coy Laboratory Products) under an atmosphere of nitrogen with oxygen less than 5 ppm and 2.5% hydrogen.

### Generation of SSN for reductive dehalogenase

The SSN was generated in July 2024 using PF13486 as a keyword with UniProt version 2024_06 (*E*-value ≤ 1e−85, 45% sequence identity, 90% represented node). Using the SSN method, we classified members within a protein family into distinct clusters, leveraging their overall sequence conservation (Fig. S1). Among the downloaded sequences, the RDases with well-defined functions formed distinct clusters (highlighted in red). Eight putative reductive dehalogenases were randomly selected from clusters with no reported dehalogenation activity (highlighted in green), including Q3ZA21, Q3Z6A6, A0A2J1DV62, A0A0C6EJ57, A0A2J1DXP0, Ros_A3X954, A0A2E6VL94, A0A0S2I3V8. The information on these uncharacterized RDases and TmrA is provided in the Supporting Information Table S4. We searched for the TAT conserved motif (RRXFXK) of these RDases and validation was performed using the InterPro web search function and multiple sequence alignment analysis. We found that Q3ZA21, Q3Z6A6, A0A2J1DV62, A0A0C6EJ57, and A0A2J1DXP0 hold a TAT signal peptide, which suggests that they are transported across the cell membrane and that they represent respiratory RDases.

### Plasmid construction and transformation of the *E. coli* host

Three *btu* plasmids were constructed (i.e., pRSFDuet-BtuCEDFB, pBADHisA-BtuCEDFB, and pBAD18-Kan-BtuCEDFB). Whereas pBADHisA and pBAD18-Kan were similar to the plasmid pBAD42 basis used by others (12, 21), the pRSFDuet-based system was used as a new system for optimization. For the pRSFDuet-BtuCEDFB plasmid construction, BtuCEDFB genes were amplified with PCR from pACYCDuet-BtuCEDFB plasmid (provided by Prof. Jiahai Zhou from Shenzhen Institute of Advanced Technology, Chinese Academy of Sciences) using primers (Btu-F: gagatataccatggtaaggaggtaattc and Btu-R: gaatgacccggtgggaagcttctatcctac) and then were constructed on the pRSFDuet plasmid using regular one-step digestion and ligation method with NcoI & Hind III restriction sites. For the construction of pBADHisA-BtuCEDFB and pBAD18-Kan-BtuCEDFB plasmids, the *btu* operon was split and amplified in two parts (*btuCED* and *btuFB*) using two sets of primers (Table S5). pBADHisA (linearized by XhoI/PstI double digestion) and pBAD18-Kan (linearized by NheI/PstI double digestion) were combined with the *btuCED* and *btuFB* gene amplicons by homologous recombinase. To enhance functional expression, the genes of interest were codon-optimized and synthesized for use in *E. coli*. Two plasmids containing the *tmrA*-gene (i.e., pET28a-TmrA and pTrcHisA-TmrA) and plasmids encoding other RDase genes (pTrcHisA-RDase) were constructed with regular one-step digestion and ligation, and the primers used are listed in Table S5. Finally, the plasmids carrying *tmrA* or other *rdhA* genes were co-transformed along with a plasmid containing the *btu* genes into *E. coli* BL21(DE3) or *E. coli* DH5a for subsequent enzyme expression. In addition, pTrcHisA-TmrA or pTrcHisA-Ros_A3X954 without pRSFDuet-BtuCEDFB plasmid was transformed into *E. coli* DH5a to investigate the role of the pRSFDuet-BtuCEDFB plasmid in the expression. The details on the combination of *TmrA*/*rdhA* plasmids, *btu* plasmid, and *E. coli* are given in Table S1.

### Expression and purification of TmrA and uncharacterized RDases

An overnight starter culture (4 mL) of either *E. coli* strain containing *tmrA*/*rdhA* plasmids with or without *btu* plasmid was inoculated into a 2-L glass bottle containing 1.8 L Terrific Broth (TB) medium with 50 μg mL^−1^ ampicillin and 50 μg mL^−1^ kanamycin sulfate and was incubated at 37°C and 180 rpm under oxic conditions. When the OD_600_ of a culture reached 0.4–0.6, 1 μM hydroxycobalamin was added to the culture, and the culture was further incubated at 37°C, 180 rpm until the OD_600_ reached 0.8–1. The culture was then placed on ice and sealed with a rubber septum and cap, and the headspace was purged with N_2_ gas for 1 h to create an anoxic environment. To this anaerobic culture, 50 mM cysteine and 50 mM ammonium ferric citrate were added, and RDase expression was induced with 1 mM isopropyl β-D-1-thiogalactopyranoside (IPTG). Noted, the plasmid pBAD-BtuCEDFB requires the addition of arabinose. The culture was incubated overnight at 15°C, 150 rpm. After 12 h, the expression culture was transferred into a Coy anaerobic glove box and kept sealed in airtight vessels for all subsequent steps. Cells were harvested by centrifugation (5,000 rpm, 10 min, 4°C), and 10 mL of lysis buffer (50 mM Tris-HCl, pH 7.5, 150 mM NaCl, 5% glycerol, 0.1% Triton X-100) was added per gram of wet cell weight. For each 10 mL of cell suspension, cells were lysed with 0.3 mg mL⁻¹ lysozyme and then disrupted using an ultrasonic disruptor in pulsed mode (5 s on, 10 s off) for a total processing time of 2 min, corresponding to 8 cycles of on/off pulses. The volumes and sonication times were scaled with the amount of cells. The resulting lysate was separated by centrifugation (16,000 rpm, 15 min, 4°C), and the supernatant was used for dehalogenation activity assays. The experimental workflow for the expression of RDase was shown in Fig. S4. The supernatant exhibiting dehalogenation activity underwent the following protein purification.

A 2-mL Ni-NTA resin column was set up inside a Coy anaerobic glove box. The column was initially washed with three column volumes of buffer A (50 mM Tris-HCl, pH 7.5), then the lysate supernatant was passed through the column, and finally two column volumes of buffer B (25 mM imidazole in buffer A) for washing. Protein elution was carried out using buffer C (250 mM imidazole in buffer A). The eluted protein fraction was dialyzed against 1 L of buffer A at 4°C for 6 h to remove imidazole, and the protein was concentrated using a 30-kDa cut-off Millipore filter tube. The concentration of the resulting protein solution was quantified using an ultra-micro volume spectrophotometer, and the solution was distributed into aliquots for flash freezing and stored in liquid nitrogen. The size of the resulting protein was analyzed by SDS-PAGE with Coomassie Brilliant Blue G250 staining (Fig. S5).

### Activity assay for dehalogenation

Organohalides, including CF, PCE, TCE, 1,1-DCA, 1,1,1-TCA, and 2,6-DBP, were selected as target compounds. All assays were carried out in 2-mL glass vials with no headspace and sealed with polytetrafluoroethylene (PTFE)-lined caps in the Coy anaerobic glove box. The reactions were carried out in the vials containing 50 mM Tris-HCl, 2 mM titanium(III) citrate, and 2 mM methyl viologen, 500 μL of cell lysate or purified RDases (0.05–0.15 μM) and organohalide (1 mM CF, PCE, TCE, 1,1-DCA, or 1,1,1-TCA; 2 mM 2,6-DBP). The final volume of the reaction was 2 mL. Lysate assays were incubated overnight at room temperature, while purified enzyme assays were incubated for 1 h at 25°C.

To stop the reactions, 1 mL of the reaction mixture was injected into a 10 mL crimp vial containing 100 μL of 1 M HCl aqueous solution through the septum with a syringe. After heating at 65°C for 10 min to ensure complete volatilization of volatile gases, headspace analysis for PCE, CF, TCE, 1,1-DCA, 1,1,1-TCA, and dechlorination products was conducted using a gas chromatograph equipped with a flame ionization detector (GC-FID). It was confirmed that no chemical was lost through the pierced septa during the preheating. For the analysis of 2,6-DBP and its debromination products, 1 mL of reaction solution was mixed with 1 mL of *n*-hexane by vortexing for 2 min, followed by centrifugation (5,000 × *g* for 8 min), and the organic layer was collected. This extraction process was repeated twice, and the combined organic extracts were subsequently desulfurized with treated copper sheets and dehydrated with heat-treated anhydrous sodium sulfate for the analysis by a gas chromatography coupled to a mass spectrometer (GC-MS).

### Effect of pH, temperature, and oxygen on dehalogenation activity of TmrA and Ros_A3X954

Unless otherwise specified, all experiments were conducted in a 2 mL reaction solution comprising 50 mM sodium phosphate buffer, 2 mM titanium(III) citrate, and 2 mM methyl viologen. The reactions contained 500 μL of purified TmrA or Ros_A3X954, along with 1 mM CF for TmrA or 2 mM 2,6-DBP for Ros_A3X954 as substrates. After mixing the components and incubating at 25°C for 1 h, residual enzyme activity was assessed by quantifying dehalogenation products according to the method mentioned above.

To investigate buffer effects on RDase activity, purified enzymes were assayed in four different buffering systems (each at 50 mM): Tris-HCl (pH range: 7.0–9.5), sodium phosphate (pH range: 6.0–8.5), HEPES (pH range: 7.0–8.0), and PIPES (pH range: 6.5–7.5). Additionally, the impact of buffer concentration on enzyme activity was evaluated using concentrations of 20 mM, 50 mM, and 100 mM.

Thermal stability was assessed by purified RDases heterologously expressed in *E. coli* DH5α with the pRSFDuet-BtuCEDFB plasmid. The purified RDases were pre-incubated at temperatures ranging from 20°C to 70°C for 5 min, followed by a 1-h reaction with the respective substrate at 25°C. For oxygen tolerance studies, purified RDase was subjected to continuous stirring under oxygenated conditions at 4°C for durations ranging from 20 to 120 min.

### Kinetic assay for the reductive dehalogenases

The kinetic constants of the purified RDases were determined with a UV spectrophotometer using varied concentrations of 2,6-DBP and CF with 2 mM of methyl viologen, 50 mM sodium phosphate buffer (pH 6.0 for Ros_A3X954 or pH 6.5 for TmrA), and 2 mM titanium(III) citrate. Methyl viologen was measured at 700 nm (*ε*_700_ = 2.4 mM^−1^ cm^−1^) (14, 38, 39). The enzymatic kinetic data were fitted to the Michaelis-Menten model to calculate *k*_cat_ and *k*_cat_/*K*_M_.

### Chemical analysis

A headspace sample of 100 μL was collected for the determination of PCE, TCE, CF, 1,1-DCA, 1,1,1-TCA, and dechlorination products using GC-FID (GC-2010, Shimazu, Kyoto, Japan) and a GS-GASPRO column (30 m × 0.32 mm × 0.25 µm; J&W Scientific, Folsom, CA, USA). Headspace samples were manually injected at a split ratio of 10:1. The initial oven temperature for GC-FID was set at 70°C for 1 min, followed by a ramp at 30°C min^−1^ to 150°C, followed by an increase at 20°C min^−1^ to 180°C, and held for 1.5 min. Helium served as the carrier gas with a column flow of 3.0 mL min^−1^. Standard curves were prepared by adding substrates into glass vials used in the activity assays, containing the same volume of buffer as described above.

2,6-DBP and its products were detected with GC-MS (QP2010 plus, Shimadzu, Japan) equipped with a Rtx-5MS capillary column (30 m × 0.25 mm × 0.26 µm) and using electron ionization in the selected ion monitoring mode (40). Helium served as the carrier gas with a flow rate of 6 mL min^ 1^ and 10:1 split ratio. The injector temperature and the ion source temperature were maintained at 250°C. The solvent delay and cutting time were both set for 2 min. An electron impact source was utilized for ionization, with the mass spectrometer scanning a range of 45 to 700 *m/z*. The oven temperature was initially set at 80°C for 1 min, increased at 10°C min^−1^ to 180°C, and held for 2 min, followed by an increase at 25°C min^−1^ to 300°C and held for 1 min.

### Analysis of cobalt, iron, and acid-labile sulfur content of the dehalogenases

The cobalt (Co) contents of the purified RDases were determined using a Perkin-Elmer 6000 ICP-MS with parameters as follows: RF power, 1,175 W; Nebulizer argon flow rate, 0.86 L min^−1^; Plasma argon flow rate, 15 L min^−1^; Auxiliary argon flow rate, 1.2 L min^−1^; Each mass integration time, 100 ms (41, 42). The purified RDases were wet-ashed in a 1:7 mixture with 65% nitric acid at 100°C for 30 min. The samples were then diluted to a final volume of 10 mL with 2% nitric acid before ICP-MS analysis (42).

The Fe content of the purified RDases was determined using the ferrozine method (26). Initially, 10 µL of protein solution was hydrolyzed by 5 µL of concentrated sulfuric acid, followed by incubation at 95°C for 30 min. Subsequently, 140 µL of an iron-zinc determination mixture, consisting of 5 mM ferrozine, 2.6% (wt/vol) ascorbic acid, and 387 mM ammonium acetate (pH 9.0), was added and mixed thoroughly. A 100 µL aliquot of the resulting solution was taken and mixed with 100 µL of water, and its absorbance was measured at 562 nm. The absorbance of a buffer solution containing protein solution at 562 nm served as the background. Employing the same method, a standard curve was generated with 0.5 to 20 nmol FeCl_3_ aqueous solution, which enabled the quantification of Fe content in the protein sample.

The content of acid-labile sulfur (S) in the protein was measured with the methylene blue assay (43). First, sulfur of proteins was released by incubating 5 µL of protein solution with 130 µL of 0.1% (m/v) zinc acetate and 5 µL of 12% NaOH for 2 h at room temperature. This led to the precipitation of sulfur as ZnS. Then, 25 µL of *N*,*N*-dimethyl-*p*-phenylenediamine (DMPD, 0.1% m/v in 5 M HCl) and 5 µL of FeCl_3_ (23 mM in 1.2 M HCl) were added to the mixture. Finally, the sample was diluted with 85 µL of water, and the absorbance at 670 nm was measured. The blank was prepared by substituting the protein solution with the corresponding buffer solution, while a solution of sodium sulfide served as the reference standard. A standard curve was established using the above method with Na_2_S aqueous solution.

### Phylogenetic tree of functionally characterized RDases

A total of 51 functionally characterized RDases, identified through transcriptional regulation analysis, native PAGE purification, or heterologous expression, were selected for phylogenetic tree construction. RDases, with epoxyqueuosine reductase (CAD7515538) serving as an outgroup, were aligned using MAFFT (v7.471, with the parameter–maxiterate set to 1,000) (44). The resulting alignments were subsequently trimmed using trimAl (v1.4.1). A maximum-likelihood phylogenetic tree of RDases was then constructed using RAxML (v8.2.12, -N 1000 -m PROTGAMMAILGX) (45, 46). The phylogenetic tree was visualized using the Interactive Tree of Life (iTOL) website (47).

## Data Availability

The accession numbers of RDases used in this study are listed in Table S4.

## SUPPLEMENTAL MATERIAL

Supplemental material aem.01980-25-s0001.docx Fig. S1 to S5; Tables S1 to S5.

Graphical abstract aem.01980-25-s0002.tif Visual depiction of the study.

ASM does not own the copyrights to Supplemental Material that may be linked to, or accessed through, an article. The authors have granted ASM a non-exclusive, world-wide license to publish the Supplemental Material files. Please contact the corresponding author directly for reuse.

## References

[1] He HZ, Li YY, Shen R, Shim H, Zeng YH, Zhao SY, Lu QH, Mai BX, Wang SQ. 2021. Environmental occurrence and remediation of emerging organohalides: a review. Environ Pollut 290:118060. doi:10.1016/j.envpol.2021.118060 34479159

[2] Justicia-Leon SD, Higgins S, Mack EE, Griffiths DR, Tang SQ, Edwards EA, Löffler FE. 2014. Bioaugmentation with distinct Dehalobacter strains achieves chloroform detoxification in microcosms. Environ Sci Technol 48:1851–1858. doi:10.1021/es403582f 24392834

[3] Benedetto Tiz D, Bagnoli L, Rosati O, Marini F, Sancineto L, Santi C. 2022. New halogen-ccontaining drugs approved by FDA in 2021: an overview on their syntheses and pharmaceutical use. Molecules 27:1643. doi:10.3390/molecules27051643 35268744 PMC8912053

[4] Gribble GW. 2024. A survey of recently discovered naturally occurring organohalogen compounds. J Nat Prod 87:1285–1305. doi:10.1021/acs.jnatprod.3c00803 38375796

[5] Gribble GW. 2023. Naturally occurring organohalogen compounds—A comprehensive review. *In* Kinghorn AD, Falk H, Gibbons S, Asakawa Y, Liu JK, Dirsch VM (ed), Naturally occurring organohalogen compounds. Springer Nature Switzerland, Cham.

[6] Hug LA, Maphosa F, Leys D, Löffler FE, Smidt H, Edwards EA, Adrian L. 2013. Overview of organohalide-respiring bacteria and a proposal for a classification system for reductive dehalogenases. Philos Trans R Soc Lond B Biol Sci 368:20120322. doi:10.1098/rstb.2012.0322 23479752 PMC3638463

[7] Leys D, Adrian L, Smidt H. 2013. Organohalide respiration: microbes breathing chlorinated molecules. Philos Trans R Soc Lond B Biol Sci 368:20120316. doi:10.1098/rstb.2012.0316 23479746 PMC3638457

[8] Lu Q, Qiu L, Yu L, Zhang S, de Toledo RA, Shim H, Wang S. 2019. Microbial transformation of chiral organohalides: distribution, microorganisms and mechanisms. J Hazard Mater 368:849–861. doi:10.1016/j.jhazmat.2019.01.103 30772625

[9] Jugder BE, Ertan H, Bohl S, Lee M, Marquis CP, Manefield M. 2016. Organohalide respiring bacteria and reductive dehalogenases: key tools in organohalide bioremediation. Front Microbiol 7:249. doi:10.3389/fmicb.2016.00249 26973626 PMC4771760

[10] Fincker M, Spormann AM. 2017. Biochemistry of catabolic reductive dehalogenation. Annu Rev Biochem 86:357–386. doi:10.1146/annurev-biochem-061516-044829 28654328

[11] Bommer M, Kunze C, Fesseler J, Schubert T, Diekert G, Dobbek H. 2014. Structural basis for organohalide respiration. Science 346:455–458. doi:10.1126/science.1258118 25278505

[12] Picott KJ, Flick R, Edwards EA. 2022. Heterologous expression of active Dehalobacter respiratory reductive dehalogenases in Escherichia coli. Appl Environ Microbiol 88:e0199321. doi:10.1128/AEM.01993-21 34851719 PMC8824204

[13] Payne KAP, Quezada CP, Fisher K, Dunstan MS, Collins FA, Sjuts H, Levy C, Hay S, Rigby SEJ, Leys D. 2015. Reductive dehalogenase structure suggests a mechanism for B_12_-dependent dehalogenation. Nature 517:513–516. doi:10.1038/nature13901 25327251 PMC4968649

[14] Miller E, Wohlfarth G, Diekert G. 1998. Purification and characterization of the tetrachloroethene reductive dehalogenase of strain PCE-S. Arch Microbiol 169:497–502. doi:10.1007/s002030050602 9575235

[15] Magnuson JK, Romine MF, Burris DR, Kingsley MT. 2000. Trichloroethene reductive dehalogenase from Dehalococcoides ethenogenes: sequence of tceA and substrate range characterization. Appl Environ Microbiol 66:5141–5147. doi:10.1128/AEM.66.12.5141-5147.2000 11097881 PMC92435

[16] Nakamura R, Obata T, Nojima R, Hashimoto Y, Noguchi K, Ogawa T, Yohda M. 2018. Functional expression and characterization of tetrachloroethene dehalogenase from Geobacter sp. Front Microbiol 9:1774. doi:10.3389/fmicb.2018.01774 30147676 PMC6095959

[17] Mac Nelly A, Kai M, Svatoš A, Diekert G, Schubert T. 2014. Functional heterologous production of reductive dehalogenases from Desulfitobacterium hafniense strains. Appl Environ Microbiol 80:4313–4322. doi:10.1128/AEM.00881-14 24814779 PMC4068680

[18] Jugder BE, Payne KAP, Fisher K, Bohl S, Lebhar H, Manefield M, Lee M, Leys D, Marquis CP. 2018. Heterologous production and purification of a functional chloroform reductive dehalogenase. ACS Chem Biol 13:548–552. doi:10.1021/acschembio.7b00846 29363941

[19] Sjuts H, Fisher K, Dunstan MS, Rigby SE, Leys D. 2012. Heterologous expression, purification and cofactor reconstitution of the reductive dehalogenase PceA from Dehalobacter restrictus. Protein Expr Purif 85:224–229. doi:10.1016/j.pep.2012.08.007 22940504

[20] Parthasarathy A, Stich TA, Lohner ST, Lesnefsky A, Britt RD, Spormann AM. 2015. Biochemical and EPR-spectroscopic investigation into heterologously expressed vinyl chloride reductive dehalogenase (VcrA) from Dehalococcoides mccartyi strain VS. J Am Chem Soc 137:3525–3532. doi:10.1021/ja511653d 25686300 PMC4516053

[21] Ng TL, Silver PA. 2024. Sustainable B_12_-dependent dehalogenation of organohalides in E. coli. ACS Chem Biol 19:380–391. doi:10.1021/acschembio.3c00585 38254247

[22] Fisher K, Halliwell T, Payne KAP, Ragala G, Hay S, Rigby SEJ, Leys D. 2023. Efficient NADPH-dependent dehalogenation afforded by a self-sufficient reductive dehalogenase. J Biol Chem 299:105086. doi:10.1016/j.jbc.2023.105086 37495113 PMC10463259

[23] Halliwell T, Fisher K, Payne KAP, Rigby SEJ, Leys D. 2021. Heterologous expression of cobalamin dependent class-III enzymes. Protein Expr Purif 177:105743. doi:10.1016/j.pep.2020.105743 32871253 PMC7585037

[24] Lanz ND, Blaszczyk AJ, McCarthy EL, Wang B, Wang RX, Jones BS, Booker SJ. 2018. Enhanced solubilization of class B radical S-adenosylmethionine methylases by improved cobalamin uptake in Escherichia coli. Biochemistry 57:1475–1490. doi:10.1021/acs.biochem.7b01205 29298049 PMC5941297

[25] Fang H, Kang J, Zhang DW. 2017. Microbial production of vitamin B_12_: a review and future perspectives. Microb Cell Fact 16:15. doi:10.1186/s12934-017-0631-y 28137297 PMC5282855

[26] Feng CX, Yan QX, Li XY, Zhao H, Huang H, Zhang XS. 2025. Discovery of a gut bacterial pathway for ergothioneine catabolism. J Am Chem Soc 147:257–264. doi:10.1021/jacs.4c09350 39700343

[27] Swingley WD, Sadekar S, Mastrian SD, Matthies HJ, Hao J, Ramos H, Acharya CR, Conrad AL, Taylor HL, Dejesa LC, Shah MK, O’huallachain ME, Lince MT, Blankenship RE, Beatty JT, Touchman JW. 2007. The complete genome sequence of Roseobacter denitrificans reveals a mixotrophic rather than photosynthetic metabolism. J Bacteriol 189:683–690. doi:10.1128/JB.01390-06 17098896 PMC1797316

[28] Paul C, Pohnert G. 2011. Production and role of volatile halogenated compounds from marine algae. Nat Prod Rep 28:186–195. doi:10.1039/c0np00043d 21125112

[29] Jacobtorweihen J, Spiegler V. 2023. Phylogenetic distribution of bromophenols in marine algae and the generation of a comprehensive bromophenol database. Phytochem Rev 22:505–542. doi:10.1007/s11101-022-09847-8

[30] Sim WJ, Lee SH, Lee IS, Choi SD, Oh JE. 2009. Distribution and formation of chlorophenols and bromophenols in marine and riverine environments. Chemosphere 77:552–558. doi:10.1016/j.chemosphere.2009.07.006 19664797

[31] Picott KJ, Edwards EA. 2024. Contrasting kinetics of highly similar chloroalkane reductive dehalogenases. Environ Sci Technol 58:22235–22244. doi:10.1021/acs.est.4c07149 39626078

[32] Rahmatullah R, Marquis CP. 2024. Evaluation of alternate hosts for recombinant expression of a reductive dehalogenase. Enzyme Microb Technol 174:110390. doi:10.1016/j.enzmictec.2023.110390 38147780

[33] Kunze C, Diekert G, Schubert T. 2017. Subtle changes in the active site architecture untangled overlapping substrate ranges and mechanistic differences of two reductive dehalogenases. FEBS J 284:3520–3535. doi:10.1111/febs.14258 28869789

[34] Molenda O, Puentes Jácome LA, Cao X, Nesbø CL, Tang S, Morson N, Patron J, Lomheim L, Wishart DS, Edwards EA. 2020. Insights into origins and function of the unexplored majority of the reductive dehalogenase gene family as a result of genome assembly and ortholog group classification. Environ Sci Process Impacts 22:663–678. doi:10.1039/c9em00605b 32159535

[35] Jugder BE, Bohl S, Lebhar H, Healey RD, Manefield M, Marquis CP, Lee M. 2017. A bacterial chloroform reductive dehalogenase: purification and biochemical characterization. Microb Biotechnol 10:1640–1648. doi:10.1111/1751-7915.12745 28631300 PMC5658581

[36] Wagner-Döbler I, Biebl H. 2006. Environmental biology of the marine Roseobacter lineage. Annu Rev Microbiol 60:255–280. doi:10.1146/annurev.micro.60.080805.142115 16719716

[37] Chen K, Huang LL, Xu CF, Liu XM, He J, Zinder SH, Li SP, Jiang JD. 2013. Molecular characterization of the enzymes involved in the degradation of a brominated aromatic herbicide. Mol Microbiol 89:1121–1139. doi:10.1111/mmi.12332 23859214

[38] Neumann A, Siebert A, Trescher T, Reinhardt S, Wohlfarth G, Diekert G. 2002. Tetrachloroethene reductive dehalogenase of Dehalospirillum multivorans: substrate specificity of the native enzyme and its corrinoid cofactor. Arch Microbiol 177:420–426. doi:10.1007/s00203-002-0409-3 11976751

[39] Thibodeau J, Gauthier A, Duguay M, Villemur R, Lépine F, Juteau P, Beaudet R. 2004. Purification, cloning, and sequencing of a 3,5-dichlorophenol reductive dehalogenase from Desulfitobacterium frappieri PCP-1. Appl Environ Microbiol 70:4532–4537. doi:10.1128/AEM.70.8.4532-4537.2004 15294782 PMC492329

[40] López P, Brandsma SA, Leonards PEG, De Boer J. 2009. Methods for the determination of phenolic brominated flame retardants, and by-products, formulation intermediates and decomposition products of brominated flame retardants in water. J Chromatogr A 1216:334–345. doi:10.1016/j.chroma.2008.08.043 18762297

[41] Peng B, Tang X, Yu C, Tan C, Yin C, Yang G, Liu Q, Yang K, Tu X. 2011. Geochemistry of trace metals and Pb isotopes of sediments from the lowermost Xiangjiang River, Hunan Province (P. R. China): implications on sources of trace metals. Environ Earth Sci 64:1455–1473. doi:10.1007/s12665-011-0969-0

[42] Liu Y, Chen LH. 1996. Simultaneous and precise determination of 40 trace elements in rock samples using ICP-MS. Geochimica 25:552–558.

[43] Rabinowitz JC. 1978. Analysis of acid-labile sulfide and sulfhydryl groups. *In* Fleischer S, Packer L (ed), Methods in enzymology. Academic Press. 10.1016/s0076-6879(78)53033-4 713838

[44] Rozewicki J, Li SL, Amada KM, Standley DM, Katoh K. 2019. MAFFT-DASH: integrated protein sequence and structural alignment. Nucleic Acids Res 47:W5–W10. doi:10.1093/nar/gkz342 31062021 PMC6602451

[45] Capella-Gutiérrez S, Silla-Martínez JM, Gabaldón T. 2009. trimAl: a tool for automated alignment trimming in large-scale phylogenetic analyses. Bioinformatics 25:1972–1973. doi:10.1093/bioinformatics/btp348 19505945 PMC2712344

[46] Stamatakis A. 2014. RAxML version 8: a tool for phylogenetic analysis and post-analysis of large phylogenies. Bioinformatics 30:1312–1313. doi:10.1093/bioinformatics/btu033 24451623 PMC3998144

[47] Letunic I, Bork P. 2024. Interactive Tree of Life (iTOL) v6: recent updates to the phylogenetic tree display and annotation tool. Nucleic Acids Res 52:W78–W82. doi:10.1093/nar/gkae268 38613393 PMC11223838

