# Supplementary material for: Developing an E. coli heterologous expression system for characterizing a marine debrominase from Roseobacter sp
Source: Appl Environ Microbiol. 2026 Apr 3;92(4):e01980-25. doi: 10.1128/aem.01980-25 (PMC13101508; doi:10.1128/aem.01980-25)
Supplement: Supplemental material — Fig. S1 to S5; Tables S1 to S5. [file aem.01980-25-s0001.docx]

**Supporting information for**

**Developing an *E. coli* Heterologous Expression System for Characterizing a Marine Debrominase from *Roseobacter* sp.**

**Summary: 14 pages**, **5 figures**, **and 5 tables.**

Xiaofang Li^1,#^, Sen Yang^2,3,#^, Yuping Liu^1^, Hua Huang^1,4^, Xinshuai Zhang^1,^*, Yin Zhong^2,5,6^*, Lorenz Adrian^7,8^, Ping’an Peng^2,5,6^

^1^Guangdong Provincial Key Laboratory of Biotechnology for Plant Development, School of Life Sciences, South China Normal University, Guangzhou 510631, China

^2^State Key Laboratory of Advanced Environmental Technology, Guangzhou Institute of Geochemistry, Chinese Academy of Sciences, Guangzhou 510640, China

^3^University of Chinese Academy of Sciences, Beijing 100049, China

^4^Shenzhen Readline Biotech CO., Ltd., Wanhe Medicine Park, Nanshan, Shenzhen, 518057, China

^5^Guangdong Key Laboratory of Environmental Protection and Resources and Utilization, Guangzhou 510640, China

^6^Guangdong-Hong Kong-Maco Joint Laboratory for Environmental Pollution and Control, Guangzhou 510640, China

^7^Department Molecular Environmental Biotechnology, Helmholtz Centre for Environmental Research – UFZ, Leipzig, Germany

^8^Chair of Geobiotechnology, Technische Universität Berlin, Berlin, Germany

^#^X. Li and S. Yang contributed equally to this work.

*Corresponding author:

Yin Zhong (E-mail: [zhongyin@gig.ac.cn](mailto:zhongyin@gig.ac.cn))

Xinshuai Zhang (E-mail: [xszhang@scnu.edu.cn](mailto:xszhang@scnu.edu.cn))

**Fig. S1.** Sequence similarity network (SSN) for PF13486.

**Fig. S2.** GC-MS chromatogram (A) and mass spectra (B, C) for debromination of 2,6-dibromophenol to 2-bromophenol by Ros_A3X954.

**Fig. S3.** Oxygen sensitivity of the heterologously expressed TmrA.

**Fig. S4.** Schematic illustration of the experimental workflow for functional expression of RDases activity.

**Fig. S5.** SDS-PAGE analysis of purified RDases.

**Table S1.** Used combinations of *E. coli* strain, RDase plasmid, and *btu* plasmid.

**Table S2.** Substrate screening of RDases.

**Table S3.** Co, Fe and acid-labile S content for RDases expressed using *E. coli* DH5α.

**Table S4.** RDases targeted in this study.

**Table S5.** Primers used for plasmid construction in this study.

**Fig. S1. Sequence similarity network (SSN) for PF13486.** Reductive dehalogenases (RDases) selected in this study are colored in green, while representative reported RDases with substrates are highlighted in red. The SSN was generated in July 2024 using PF13486 as key word with UniProt version 2024_06 (1,473 members, thresholds: e^-85^, 45% sequence identity, 90% represented node).

**Fig. S2. GC-MS chromatogram (A) and mass spectra (B, C) for debromination of 2,6-dibromophenol to 2-bromophenol by Ros_A3X954.**

**Fig. S3. Oxygen sensitivity of the heterologously expressed TmrA.** Shown is the production of DCM after incubation of 1 mM CF for 1 h with 500 μL of purified TmrA (0.05 μM) that had been exposed to air for a defined period of time. TmrA was heterologously expressed in *E. coli* DH5α with the pRSFDuet-BtuCEDFB plasmid. Data from three independent replicates are presented.

**Fig. S4. Schematic illustration of the experimental workflow for functional expression of RDases activity.**

**Fig. S5. SDS-PAGE analysis of purified RDases.** (A) Btu and TmrA (~53 kDa). M: Protein ladder; 1: Supernatant; 2: Flow-through; 3: Wash fraction; 4: Pellet; 5–9: Protein elution fractions from tubes 1 to 5, respectively. (B) Btu and Ros_A3X954 (~48 kDa). All proteins in the Btu pathway were His-tagged. From the gel image, BtuC (~37 kDa) is also observed, illustrating the successful purification process of recombinant protein BtuC using Ni-NTA resin.

**Table S1. Used combinations of *E. coli* strain, RDase plasmid, and *btu* plasmid.**

| **Group** | ***E. coli*** | **RDase plasmid** | **Btu plasmid** |
| --- | --- | --- | --- |
| 1 | BL21 (DE3) | pET28a-TmrA | pBADHisA-BtuCEDFB |
| 2 | BL21 (DE3) | pTrcHisA-TmrA | pBAD18-Kan-BtuCEDFB |
| 3 | BL21 (DE3) | pTrcHisA-TmrA | pRSFDuet-BtuCEDFB |
| 4 | BL21 (DE3) | pTrcHisA-Q3ZA21 | pRSFDuet-BtuCEDFB |
| 5 | BL21 (DE3) | pTrcHisA-Q3Z6A6 | pRSFDuet-BtuCEDFB |
| 6 | BL21 (DE3) | pTrcHisA-A0A2J1DV62 | pRSFDuet-BtuCEDFB |
| 7 | BL21 (DE3) | pTrcHisA-A0A0C6EJ57 | pRSFDuet-BtuCEDFB |
| 8 | BL21 (DE3) | pTrcHisA-A0A2J1DXP0 | pRSFDuet-BtuCEDFB |
| 9 | BL21 (DE3) | pTrcHisA-Ros_A3X954 | pRSFDuet-BtuCEDFB |
| 10 | BL21 (DE3) | pTrcHisA-A0A2E6VL94 | pRSFDuet-BtuCEDFB |
| 11 | BL21 (DE3) | pTrcHisA-A0A0S2I3V8 | pRSFDuet-BtuCEDFB |
| 12 | DH5α | pTrcHisA-TmrA | pRSFDuet-BtuCEDFB |
| 13 | DH5α | pTrcHisA-TmrA | **–** |
| 14 | DH5α | pTrcHisA-Ros_A3X954 | pRSFDuet-BtuCEDFB |
| 15 | DH5α | pTrcHisA-Ros_A3X954 | **–** |
| 16 | DH5α | pTrcHisA-Q3ZA21 | pRSFDuet-BtuCEDFB |
| 21 | DH5α | pTrcHisA-Q3Z6A6 | pRSFDuet-BtuCEDFB |
| 17 | DH5α | pTrcHisA-A0A2J1DV62 | pRSFDuet-BtuCEDFB |
| 18 | DH5α | pTrcHisA-A0A0C6EJ57 | pRSFDuet-BtuCEDFB |
| 22 | DH5α | pTrcHisA-A0A2J1DXP0 | pRSFDuet-BtuCEDFB |
| 19 | DH5α | pTrcHisA-A0A2E6VL94 | pRSFDuet-BtuCEDFB |
| 20 | DH5α | pTrcHisA-A0A0S2I3V8 | pRSFDuet-BtuCEDFB |

**–**: Without Btu plasmid.

**Table S2. Substrate screening of RDases.**

| Uniprot ID | PCE | 1,1-DCA | CF | 1,1,1-TCA | TCE | 2,6-DBP |
| --- | --- | --- | --- | --- | --- | --- |
| TmrA (WP_034377773) | × | × | √ | × | × | × |
| Q3ZA21 | × | × | × | × | × | × |
| Q3Z6A6 | × | × | × | × | × | × |
| A0A2J1DV62 | × | × | × | × | × | × |
| A0A0C6EJ57 | × | × | × | × | × | × |
| A0A2J1DXP0 | × | × | × | × | × | × |
| Ros_A3X954 | × | × | × | × | × | √ |
| A0A2E6VL94 | × | × | × | × | × | × |
| A0A0S2I3V8 | × | × | × | × | × | × |

^√^: Dehalogenating activity was detected; ^×^: No dehalogenating activity was detected.

**Table S3. Co, Fe and acid-labile S content for RDases expressed using *E. coli* DH5α.**

| RDase plasmid | BtuCEDFB plasmid | Co (mol per mol enzyme) | Fe (mol per mol enzyme) | Acid-labile S (mol per mol enzyme) |
| --- | --- | --- | --- | --- |
| pTrcHisA-TmrA | – | 0.35 ± 0.003 | 4.40 ± 0.05 | 3.35 ± 0.003 |
| pTrcHisA-TmrA | BtuCEDFB plasmid | 0.83 ± 0.01 | 9.52 ± 0.03 | 10.70 ± 0.06 |
| pTrcHisA-Ros_A3X954 | – | 0.06 ± 0.0007 | 3.51 ± 0.04 | 3.88 ± 0.007 |
| pTrcHisA-Ros_A3X954 | BtuCEDFB plasmid | 0.11 ± 0.003 | 8.06 ± 0.8 | 5.64 ± 0.8 |

**–**: Without Btu plasmid.

**Table S4. RDases targeted in this study.**

| RDase | Accession ID | Organism | RDase sequence length (aa)^a^ | TAT (start:end)^b^ |
| --- | --- | --- | --- | --- |
| TmrA | WP_034377773 | *Dehalobacter* sp. UNSWDHB | 455 | 1:39 |
| A0A2J1DXP0 | A0A2J1DXP0 | *Dehalococcoides mccartyi* | 515 | 1:40 |
| A0A2J1DV62 | A0A2J1DV62 | *Dehalococcoides mccartyi* | 506 | 1:36 |
| Q3Z6A6 | Q3Z6A6 | *Dehalococcoides mccartyi* 195 | 500 | 1:31 |
| Q3ZA21 | Q3ZA21 | *Dehalococcoides mccartyi* 195 | 455 | 1:34 |
| A0A0C6EJ57 | A0A0C6EJ57 | *Dehalococcoides* sp. UCH007 | 532 | 1:66 |
| Ros_A3X954 | A3X954 | *Roseobacter* sp. MED193 | 395 | No |
| A0A0S2I3V8 | A0A0S2I3V8 | *Salinivirga cyanobacteriivorans* | 453 | No |

*^a^*: length of RDase sequence with TAT signal peptide.

*^b^*: start and end position of the TAT signal peptide in the RDase sequence.

**Table S5. Primers used for plasmid construction in this study. Restriction sites are shown in bold and underlined.**

| Plasmid | Primers seq 5' to 3' |
| --- | --- |
| pET28a-TmrA | TmrA- NdeⅠ-F:gtg ccg cgc ggc agc **cat atg** gac aag gaa aaa ag  TmrA-XhoⅠ-R: gca gcc gga tc**c tcg ag**t tat ttc cac caa tct gc |
| pTrcHisA-TmrA | TmrA-XhoⅠ-F: gat ggg gat ccg ag**c tcg ag**a tgg aca agg aaa aaa gta aca ac  TmrA-HindⅢ-R: ccg cca aaa cag cc**a agc tt**t tat ttc cac caa tct gct ttc gct |
| pTrcHisA-Hem_B0TD77 | Hem_B0TD77- XhoⅠ-F: ggt gcc gcg cgg c**ct cga g**at gat ccc gcg tg  Hem_B0TD77-HindⅢ-R: gca gcc gga tc**a agc tt**t tac agg ccc tg |
| pTrcHisA-Ros_A3X954 | Ros_A3X954-XhoⅠ-F: ggt gcc gcg cgg c**ct cag a**at gag c  Ros_A3X954-HindⅢ-R: gca gcc cgt aa**a agc tt**c acc acc acc |
| pTrcHisA-Q3ZA21 | Q3ZA21- XhoⅠ-F: gtg ccg cgc ggc **ctc gag** atg gat g  Q3ZA21- HindⅢ-R: ctt tgt tag cag ccg gat c**aa gct t**tt aac |
| pTrcHisA-A0A2J1DV62 | A0A2J1DV62- XhoⅠ-F: ggt gcc gcg cgg c**ct cga g**at gag tca gtt tc  A0A2J1DV62- HindⅢ-R: gtt agc agc cgg atc **aag ctt** tta ctg ggt g |
| pTrcHisA-A0A0C6EJ57 | A0A0C6EJ57- XhoⅠ-F: gtg ccg cgc ggc **ctc gag** atg ctg aat ggc tttc  A0A0C6EJ57- HindⅢ-R: gtt agc agc cgg atc **aag ctt** tta ctg |

**Table S5. Primers used for plasmid construction in this study. Restriction sites are shown in bold and underlined. (continued)**

| Plasmid | Primers seq 5' to 3' |
| --- | --- |
| pTrcHisA-A0A2E6VL94 | A0A2E6VL94- XhoⅠ: ggt gcc gcg cgg c**ct cga g**at ggt tct g  A0A2E6VL94-R- HindⅢ: ggt ggt ggt ggt g**aa gct t**tt att tc |
| pTrcHisA-A0A0S2I3V8 | A0A0S2I3V8 - XhoⅠ: ggt gcc gcg cgg c**ct cga g**at gta cct g  A0A0S2I3V8-R- HindⅢ: ggt ggt ggt g**aa gct t**tt aac ttt tgg cc |
| pTrcHisA-Q3Z6A6 | Q3Z6A6-XhoⅠ: ggt gcc gcg cgg cag c**ct cga g**cc ggt gtt tc  Q3Z6A6-HindⅢ: ggt ggt ggt ggt g**aa gct t**tt att tac c |
| pTrcHisA-A0A2J1DXP0 | A0A2J1DXP0-XhoⅠ: ggt gcc gcg cgg c**ct cga g**at gag tgc att tc  A0A2J1DXP0-HindⅢ: ggt ggt ggt ggt g**aa gct t**tt aca gat tg |
| pRSFDuet-BtuCEDFB | Btu-F(NcoI): gag ata ta**c cat gg**t aag gag gta att c  Btu-R(HindIII): gaa tga ccc ggt ggg **aag ctt** cta tcc tac |
| pBADHisA-BtuCEDFB | BtuCED-F (XhoⅠ): gat ggg gat ccg ag**c tcg ag**a tgc tga cac ttg ccc gc  BtuCED-R: cag tga ctt agc cat cag atg gtc gaa atc agc att ct  BtuFB-F: gat ttc gac cat ctg atg gct aag tca ctg ttc agg g  BtuFB-R(PstⅠ): gaatgacccggtggg**ctgcag**ctatcctac |

**Table S5. Primers used for plasmid construction in this study. Restriction sites are shown in bold and underlined. (continued)**

| Plasmid | Primers seq 5' to 3' |
| --- | --- |
| pBAD18-Kan-BtuCEDFB | BtuCED-F (NheI): acc cgt ttt ttt gg**g cta gc**a tgc tga cac ttg ccc gc  BtuCED-R: cag tga ctt agc cat cag atg gtc gaa atc agc att ct  BtuFB-F: gat ttc gac cat ctg atg gct aag tca ctg ttc agg g  BtuFB-R (PstⅠ): gct tgc atg c**ct gca g**tc aga agg tgt agc tgc cag a |
