# Supplementary figures and images for: Developing an E. coli heterologous expression system for characterizing a marine debrominase from Roseobacter sp
Source: Appl Environ Microbiol. 2026 Apr 3;92(4):e01980-25. doi: 10.1128/aem.01980-25 (PMC13101508; doi:10.1128/aem.01980-25)

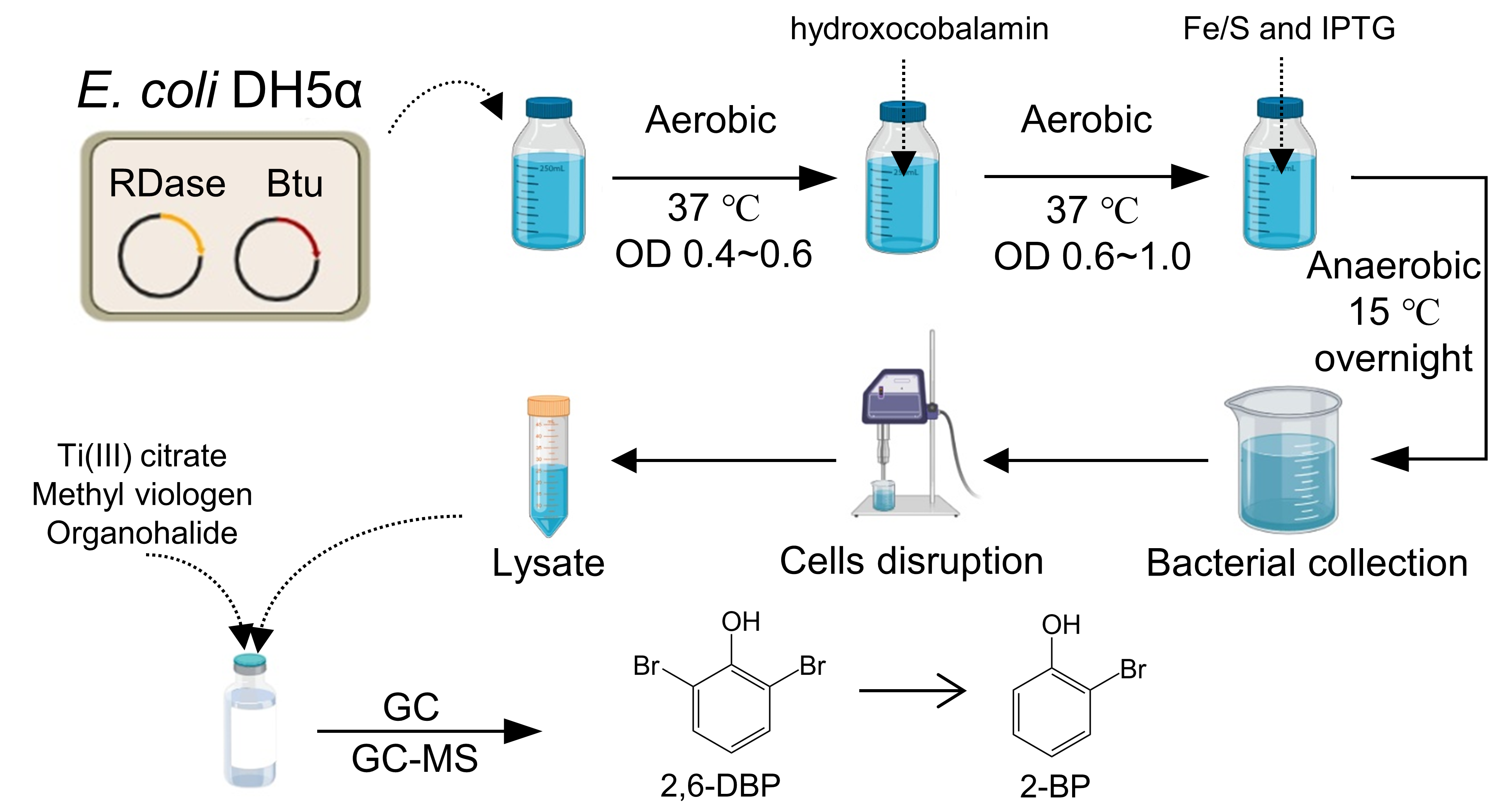

Supplement: Graphical abstract — Visual depiction of the study. [file aem.01980-25-s0002.tif]
